# Apoptosis induction and tumor cell repopulation: The yin and yang of radiotherapy

**DOI:** 10.1186/1748-717X-6-176

**Published:** 2011-12-19

**Authors:** Kirsten Lauber, Luis E Munoz, Christian Berens, Verena Jendrossek, Claus Belka, Martin Herrmann

**Affiliations:** 1 Department of Radiation Oncology, LMU Munich, Germany; 2 Department of Internal Medicine 3, Friedrich-Alexander-University Erlangen-Nuremberg, Germany; 3 Department of Biology, Friedrich-Alexander-University Erlangen-Nuremberg, Germany; 4 Institute of Cell biology, University Hospital Essen, Germany

## 

The induction of tumor cell death is a central goal of radiotherapy. Surprisingly, a recent study sheds new light on this process, and the results presented by Huang et al. strongly question the benefit of radiation-induced apoptosis for the outcome of cancer radiotherapy.

In their study, Huang and coworkers describe that induction of apoptosis by radiotherapy stimulates rapid tumor cell repopulation - a process crucially dependent on caspase 3 activity [1]. These findings are of immense relevance for the clinical use of radiotherapy, particularly when combined with targeted agents aiming at radiosensibilization and enhanced apoptosis induction [2-8]. In contrast to the results presented by Huang et al., previous work has convincingly demonstrated that the increased induction of apoptotic cell death, for example by the combination of agonistic TRAIL antibodies with radiation, results in a pronounced benefit for long term tumor control in a colorectal xenograft model [9-11]. Parallel *in vitro *studies have revealed that enhanced caspase-mediated apoptosis is the underlying mechanism for the improved eradication of clonogenic tumor cells [10,12]. Thus, the effect described by Huang and coworkers should be considered as a repopulation mechanism, which is of importance under specific, currently unknown circumstances. In this regard, it can be speculated that the balance between the apoptotic net cell kill, and the PGE_2_-driven tumor cell survival and repopulation accounts for the reported discrepancies.

Nevertheless, the study by Huang and coworkers is highly interesting, in particular because of the elegant experiments, with which the signaling cascade of apoptosis-induced tumor cell repopulation was unraveled. The downstream mechanisms identified by Huang et al. involve the caspase 3-dependent cleavage and activation of iPLA_2 _and the subsequent production of PGE_2_. During radiation-induced cell death *in vitro *PGE_2 _was shown to be released by tumor cells as well as by fibroblasts. *In vivo *(in experimental mouse models), both tumor and tumor stroma cells reportedly contributed to rapid tumor cell repopulation by few residual tumor cells in response to radiotherapy.

We would like to point out that the tumor stroma contains a highly interesting cell population, which might contribute to or even dominate the tumor-growth-stimulating PGE_2 _production: macrophages that govern the elimination of apoptosing cells and instigate tissue healing by producing a clearance-related cytokine milieu, including PGE_2 _[13-15]. Of note, Huang and coworkers observed that more macrophages were present in irradiated (apoptotic) tumors than in non-irradiated ones. These phagocytes have presumably been recruited by apoptotic cell-derived find-me signals, such as nucleotides and lysophosphatidylcholine, which - akin to PGE_2 _- are released in a caspase 3- or caspase 3- plus iPLA_2_-dependent manner, respectively [16-19]. Caspase 3 apparently is a key player in this context. So it should be taken into consideration that caspase 3 controls more processes than the release of PGE_2 _or phagocyte-recruiting attraction signals by apoptotic cells. Caspase 3 also shifts the balance between apoptosis, necrosis and autophagy as described by Huang et al., and orchestrates the central features of apoptosis, which have profound impact on macrophage activation and cytokine production after the engulfment of apoptotic cells. As such, externalization of phosphatidylserine, bleb formation and internucleosomal DNA fragmentation, known to be crucial for the subsequent anti-inflammatory cytokine production by macrophages [20-22], have been reported to depend on caspase 3 activity during apoptosis [23-27]. Thus, caspase 3-positive apoptosing cells recruit more macrophages, are more efficiently phagocytosed, and induce a stronger anti-inflammatory, wound-healing and growth promoting phagocyte response, including PGE_2 _production, than their caspase 3-negative counterparts. This might contribute or translate to the clinical observation by Huang et al. that elevated expression levels of activated caspase-3 were associated with a poor outcome in two patient cohorts with head and neck carcinoma or with advanced stage breast carcinoma.

**Figure 1:**
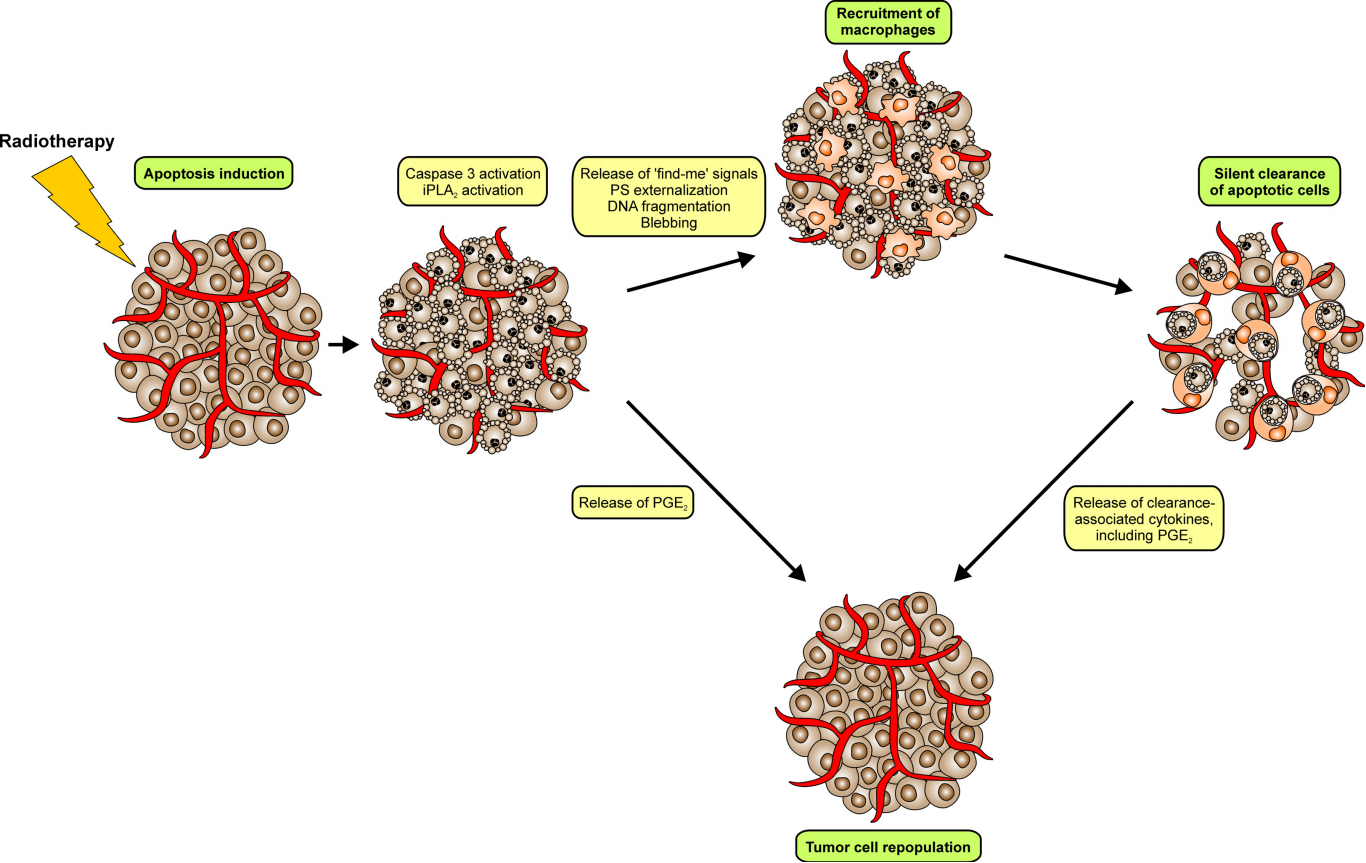
**Radiotherapy-induced apoptosis leads to caspase 3-dependent tumor cell repopulation**.

Overall, Huang and coworkers suggest a scenario, in which the caspase 3-driven iPLA_2_-dependent PGE_2 _production by irradiated tumor and tumor stroma cells plays a pivotal role for radiation-induced tumor cell repopulation and for poor therapeutic outcome. We would like to add the clearance of apoptosing cells by macrophages, and the subsequently produced clearance-related anti-inflammatory milieu, including PGE_2_, to this model (Figure 1). Intriguingly, in their final step both processes rely on cyclooxygenase activity, thus re-opening the therapeutic perspective of cautious cyclooxygenase inhibition as an adjuvant to radiotherapy [28-30]. Up to now several clinical trials have documented that a safe combination of cyclooxygenase inhibitors (celecoxib) and radiotherapy is feasible, yet most of the trials were not adequately powered to detect meaningful differences in tumor control [31,32]. Thus, further clinical trials, in particular phase III studies, are required to shed light onto this issue. In the same line, it should be addressed, whether caspase inhibition (upstream of PGE_2 _production) in combination with radiotherapy displays a benefit for the overall therapeutic outcome - provided that tumor cell systems utilizing the caspase 3-dependent, PGE_2_-driven tumor cell repopulation can reliably be identified.
